# Genome Sequences of Mycobacteriophages Amgine, Amohnition, Bella96, Cain, DarthP, Hammy, Krueger, LastHope, Peanam, PhelpsODU, Phrank, SirPhilip, Slimphazie, and Unicorn

**DOI:** 10.1128/genomeA.01202-17

**Published:** 2017-12-07

**Authors:** Kirk R. Anders, Nazir Barekzi, Aaron A. Best, Gregory D. Frederick, Dmitri V. Mavrodi, Edwin Vazquez, SEA-PHAGES, Nana Yaa A. Amoh, Frederick N. Baliraine, William J. Buchser, Thomas P. Cast, Carmen E. Chamberlain, Hui-Min Chung, William A. D’Angelo, Christian T. Farris, Mariceli Fernandez-Martinez, Haley D. Fischman, Mark H. Forsyth, Anna G. Fortier, Kara F. Gallo, Greta J. Held, Miguel A. Lomas, Natalia Y. Maldonado-Vazquez, Claudia H. Moonsammy, Peace Namboote, Sudip Paudel, Sarah-Elizabeth M. Polley, Gabriella M. Reyes, Michael R. Rubin, Margaret S. Saha, Joseph Stukey, Tristan D. Tobias, Rebecca A. Garlena, Ty H. Stoner, Steven G. Cresawn, Deborah Jacobs-Sera, Welkin H. Pope, Daniel A. Russell, Graham F. Hatfull

**Affiliations:** a Department of Biology, Gonzaga University, Spokane, Washington, USA; b Department of Biology, Norfolk State University, Norfolk, Virginia, USA; c Department of Biology, Hope College, Holland, Michigan, USA; d Department of Biology and Kinesiology, LeTourneau University, Longview, Texas, USA; e Department of Biological Sciences, The University of Southern Mississippi, Hattiesburg, Mississippi, USA; f University of Puerto Rico at Cayey, Cayey, Puerto Rico; g Department of Biology, College of William and Mary, Williamsburg, Virginia, USA; h Department of Biology, University of West Florida, Pensacola, Florida, USA; i Department of Biological Sciences, University of Pittsburgh, Pittsburgh, Pennsylvania, USA; j Department of Biology, James Madison University, Harrisonburg, Virginia, USA

## Abstract

We report the genome sequences of 14 cluster K mycobacteriophages isolated using *Mycobacterium smegmatis* mc²155 as host. Four are closely related to subcluster K1 phages, and 10 are members of subcluster K6. The phage genomes span considerable sequence diversity, including multiple types of integrases and integration sites.

## GENOME ANNOUNCEMENT

The increase in student engagement in authentic research experiences and decreasing cost of DNA sequencing has led to more than 1,000 mycobacteriophage genomes being sequenced through the Science Education Alliance–Phage Hunters Advancing Genomics and Evolutionary Science (SEA-PHAGES) program (1). The genomes reveal diverse genomic architecture and have been grouped into more than 30 clusters and singletons based on sequence similarity (2). We report the genome sequences of four subcluster K1 (Bella96, LastHope, Peanam, and Slimphazie) and 10 subcluster K6 mycobacteriophages (Amgine, Amohnition, Cain, DarthP, Hammy, Krueger, PhelpsODU, Phrank, SirPhilip, and Unicorn) isolated on the host strain *Mycobacterium smegmatis* mc²155 by enrichment between 25 and 37°C. Transmission electron microscopy reveals that they all have a siphoviral morphology.

Genomes were sequenced using Illumina MiSeq v3 chemistry (150-cycle, single-end reads) and assembled using Newbler and Consed, resulting in coverages of at least 300-fold. Genes were predicted using DNA Master (http://cobamide2.bio.pitt.edu/), Glimmer (3), GeneMark (4), ARAGORN (5), tRNAscan-SE (6), and Starterator (https://github.com/SEA-PHAGES/starterator). The K1 genomes range in size from 60,143 to 61,812 bp and in GC contents from 66.1 to 68.5% the K6 genomes range from 56,580 to 62,236 bp in length and in GC contents from 66.1 to 67.2%. All genomes contain defined ends with 3′ extensions of 10 or 11 bases with variation at two positions in the otherwise conserved sequence (Table 1).

**TABLE 1:** Newly sequenced mycobacteriophage genome sequences

Name	Cluster	GenBank accession no.	Genome length (bp)	G+C contents (%)	Fold coverage	No. of tRNAs	No. of CDS^a^	End sequence	Isolation location
Bella96	K1	MF377440	60,746	66.1	344	1	97	CTCGTAGGCAT	Grand Ledge, MI
LastHope	K1	MF140416	60,934	66.9	854	1	101	CTCGTAGGCAT	Juana Diaz, PR
Peanam	K1	MF185722	61,041	68.5	2,198	1	99	CTCGTAGGCAT	Longview, TX
Slimphazie	K1	MF140428	60,143	66.6	2,516	1	99	CTCGTAGGCAT	Pensacola, FL
Amgine	K6	MF324915	62,236	66.4	1,081	0	97	CTCGTAGGCAT	Constantine, MI
Amohnition	K6	MF140398	61,761	67.2	1,272	1	95	CTCGTAGGCAT	Williamsburg, VA
Cain	K6	MF324913	60,813	66.3	1,621	2	100	CTCGGGGCAT	Spokane, WA
DarthP	K6	MF140406	61,594	67.2	3,102	1	95	CTCGTAGGCAT	Pensacola, FL
Hammy	K6	KY087993	61,812	67.2	2,914	1	95	CTCGTAGGCAT	Slidell, LA
Krueger	K6	MF324914	60,321	66.5	314	1	97	CTCGGGGCAT	Holland, MI
PhelpsODU	K6	MF324909	56,580	66.1	1,868	2	90	CTCGGGGCAT	Suffolk, VA
Phrank	K6	MF324912	61,109	66.2	2,442	2	101	CTCGGGGCAT	Spokane, WA
SirPhilip	K6	MF324911	61,882	66.7	2,118	1	97	CTCGTAGGCAT	Spokane, WA
Unicorn	K6	MF324908	61,208	66.2	986	2	100	CTCGGGGCAT	Suffolk, VA

a CDS, coding sequences.

Putative gene functions were assigned using BLASTp (7), HHpred (8), and Phamerator (9). The genomes have 90 to 101 protein-coding genes and 1 or 2 tRNAs, with the exception of Amgine, which has none. The proportion of genes with predicted functions range between 34 and 58%. Based on nucleotide similarity and shared gene content, Bella96, Slimphazie, LastHope, and Peanam were assigned to subcluster K1, while the remaining 10 phages were identified as members of subcluster K6 (2, 10).

All of the phage genomes have generally well conserved functions and gene order, although the subcluster K6 genomes are more diverse at the nucleotide level than the K1 genomes. The putative lysis cassette consisting of lysin A, lysin B, and holin genes are conserved in subclusters K1 and K6, but the adjacent region extending through the integration cassette shows considerable diversity among the cluster K phages (11). LastHope is relatively rare among cluster K phages in coding for a serine integrase (Int-S), and its nearest relative is the Int-S of *Streptomyces* phage phiCAM (37% amino acid identity) (2); most (90%) cluster K phages code for tyrosine integrases. The putative *attP* sites of DarthP and Hammy partially overlap an *M. smegmatis* tRNA-Lys^(CTT)^ gene (MSMEG_4746), while Krueger, Amohnition, Amgine, and SirPhilip overlap a tRNA-Lys^(TTT)^ (MSMEG_5758) (12). Interestingly, Phrank, PhelpsODU, Cain, and Unicorn have both putative *attP* sites and may be able to integrate into either *attB* site. In contrast, the predicted chromosomal *attB* site used by Bella96, Slimphazie, and Peanam is located within the 3′ end of a host transfer-messenger RNA (tmRNA) as in some other cluster K phages (11). The subcluster K6 genomes show variability in the numbers and types of genes in their right arms (between *int* and rightmost *cos*).

### Accession number(s).

Nucleotide sequence accession numbers are listed in Table 1.
